# Complete Genome Sequence of the Multidrug-Resistant Pseudomonas aeruginosa Endemic Houston-1 Strain, Isolated from a Pediatric Patient with Cystic Fibrosis and Assembled Using Oxford Nanopore and Illumina Sequencing

**DOI:** 10.1128/MRA.00903-19

**Published:** 2019-10-24

**Authors:** Jennifer K. Spinler, Sabeen Raza, Jessica K. Runge, Ruth Ann Luna

**Affiliations:** a Department of Pathology & Immunology, Baylor College of Medicine, Houston, Texas, USA; b Texas Children’s Microbiome Center, Department of Pathology, Texas Children’s Hospital, Houston, Texas, USA; Loyola University Chicago

## Abstract

Hybrid *de novo* assembly of Illumina/Nanopore sequence data produced complete circular sequences of the chromosome and a plasmid for the multidrug-resistant Pseudomonas aeruginosa Houston-1 strain. This provides a high-quality representative sequence for a lineage endemic to a pediatric cystic fibrosis care center at Texas Children’s Hospital.

## ANNOUNCEMENT

Chronic respiratory infections with multidrug-resistant Pseudomonas aeruginosa (MDR-PA) result in greater morbidity and mortality in patients with cystic fibrosis (CF). An MDR-PA strain, Houston-1, identified in 2004 as endemic in patients with CF at Texas Children’s Hospital in Houston, TX, was associated with increased patient morbidity (1) and continues to persist today. We report the complete sequence of the MDR-PA Houston-1 (HOU1) genome and its native plasmid, pHOU1-1, to serve as a high-quality reference sequence for this endemic lineage.

The HOU1 strain was isolated from a patient sputum specimen by aerobic culture on blood agar at 37°C, identified as P. aeruginosa by morphological distinction on multiple media (blood agar, chocolate agar, MacConkey agar, BC [Burkholderia cepacia] agar, Columbia agar with colistin and nalidixic acid [CNA agar], and CHROMagar Staph aureus), and stored at −80°C. Antibiotic susceptibility testing for MDR-PA confirmation was performed by disk diffusion on Mueller-Hinton plated medium and defined as being resistant to all antibiotics that were routinely evaluated in two or more of the following groups: aminoglycosides (tobramycin, gentamicin, and amikacin), fluoroquinolones (ciprofloxacin), and beta-lactams (ceftazidime, meropenem, piperacillin, ticarcillin-clavulanate, and aztreonam). A cryofrozen isolate of the original HOU1 stock was streaked for isolation onto LB agar and incubated at 37°C for 18 h, and a single colony was subcultured in LB broth under the same conditions. Genomic DNA extracted using the MasterPure complete DNA and RNA purification kit (catalog number MC85200; Lucigen) was used for both Illumina and Nanopore sequencing. DNA was fragmented and libraries prepared for Illumina sequencing using the NEXTflex Rapid DNA-seq kit (catalog number NOVA-5149-02; Bioo Scientific). The genome was confirmed to be P. aeruginosa by 16S rRNA gene sequencing using the NEXTflex 16S V4 Amplicon-Seq kit 2.0 on the Illumina MiSeq platform, followed by operational taxonomic unit (OTU) clustering with UPARSE (2) and taxonomic assignment with the RDP Classifier and SILVA 16S rRNA databases (3). Illumina paired-end reads (1,158,342 2 × 150-bp reads) were generated on the MiSeq platform (Illumina, San Diego, CA, USA) using the Illumina MiSeq reagent kit v2 (catalog number MS-102-2002) and PhiX control kit v3 (catalog number FC-110-3001). Default parameters were used for all software, unless otherwise specified. Raw reads were quality filtered and adapter trimmed with Trimmomatic v0.36 (4). Oxford Nanopore Technologies (ONT) sequencing libraries were prepared using the manufacturer’s rapid barcoding kit (SQK-RBK004), and sequencing was carried out on a MinION device using flow cell type R9.4.1 (FLO-MIN106D) without fragmentation. Guppy v3.1.5 was used to base call, quality filter (minimum Q score, 10), demultiplex, barcode, and quality trim the 1.106 Gbp in 108,733 ONT-passed reads (*N*_50_, 16,879 bp). Nanopore and Illumina reads were hybrid *de novo* assembled using Unicycler v0.4.8-beta (5) in the normal assembly mode. Pilon v1.23 (6) completed 10 polishing cycles and corrected 121 single nucleotide polymorphisms, 10 small insertions totaling 10 bases, and 2 small deletions totaling 2 bases. Unicycler reported one circular chromosome of 6,123,373 bp (66.4% G+C content) and a single circular 167,069-bp plasmid. BBMap v38.68 (https://sourceforge.net/projects/bbmap/) calculated the average coverages for the chromosome (47.7× for short reads, 60.2× for long reads) and plasmid (98.5× for short reads, 130.7× for long reads). Annotations with the NCBI Prokaryotic Genome Annotation Pipeline (7) indicate 5,780 protein-coding genes, 12 rRNA operons, 64 tRNAs, 4 noncoding RNAs (ncRNAs), and 3 CRISPR arrays in the chromosome, with 167 protein-coding genes and 7 tRNAs in the plasmid.

### Data availability.

The genome (GenBank accession number CP042269) and plasmid (GenBank accession number CP042268) sequences for HOU1 were deposited in the NCBI under BioProject number PRJNA556762. The Illumina paired-end fastq and ONT base-called fastq files are available in the Sequence Read Archive under accession numbers SRX6596145 and SRX6596144, respectively.
